# Synthesis and characterization of novel protein nanodots as drug delivery carriers with an enhanced biological efficacy of melatonin in breast cancer cells†

**DOI:** 10.1039/d0ra08959a

**Published:** 2021-03-01

**Authors:** Kanchan Yadav, Megha Das, Nurul Hassan, Archana Mishra, Jayeeta Lahiri, Ashutosh Kumar Dubey, Sanjeev Kumar Yadav, Avanish Singh Parmar

**Affiliations:** Department of Physics, Indian Institute of Technology (BHU) Varanasi-221005 India asparmar.phy@itbhu.ac.in; Department of Zoology, Institute of Science, BHU Varanasi India; Department of Physics, University of Hyderabad Hyderabad India; Nuclear Agriculture and Biotechnology Division, Bhabha Atomic Research Centre Mumbai India; Department of Physics, Banaras Hindu University Varanasi India; Department of Ceramic Engineering, Indian Institute of Technology (BHU) Varanasi India

## Abstract

Melatonin is a potent antioxidant, chemotherapeutic and chemo preventive agent against breast cancer. However, its short half-life is one of the major limitations in its application as a therapeutic drug. To overcome this issue, the green-emitting protein nanodot (PND) was synthesized by a one-step hydrothermal method for loading melatonin. The synthesized pH-7 and pH-2 PND showed a quantum yield of 22.1% and 14.0%, respectively. The physicochemical characterization of both PNDs showed similar morphological and functional activities. Furthermore, the biological efficacy of melatonin-loaded PND (MPND) was evaluated in a breast cancer cell line (MDA-MB-231) for live-cell imaging and enhanced nano-drug delivery efficacy. Interestingly, the permeability of neutral pH PND in both cell cytoplasm and nucleus nullifies the limitations of real-time live-cell imaging, and ensures nuclear drug delivery efficacy. Neutral pH PND showed better cell viability and cytotoxicity as a fluorescence bioimaging probe compared to acidic PND. The bioavailability and cell cytotoxicity effect of MPND on MDA-MB-231 breast cancer cells were studied through confocal and migration assay. Results showed that MPND causes enhanced bioavailability, better cellular uptake, and inhibition of the migration of breast cancer cells as compared to the drug alone. Besides, the synthesized MPND showed no sign of fluorescence quenching even at a high concentration of melatonin, making it an ideal nanocarrier for bioimaging and drug delivery.

## Abstract 2

A novel nanodot-using protein has been synthesized for the live cell imaging and drug delivery of melatonin in breast cancer cells. Its unique properties hold potential for various biomedical applications in the field of bioimaging and drug delivery.

## Graphical abstract

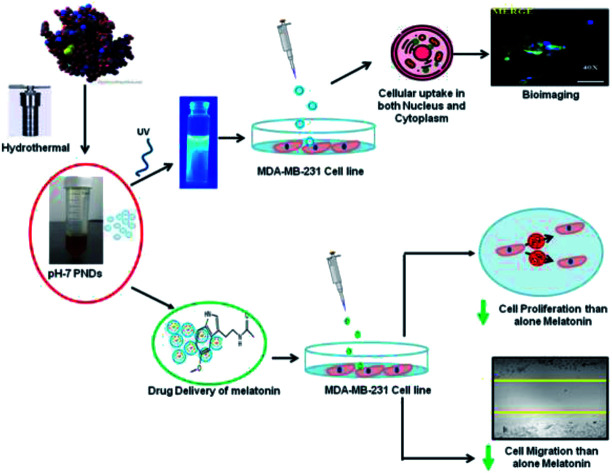

## 1. Introduction

Melatonin (*N*-acetyl-5-methoxytryptamine) (Mel) is an indolic compound with diverse physiological functions.^1^ This natural compound is produced primarily from the pineal gland of humans and other mammals, and controls the circadian rhythm and sleep–wake cycle, growth and development of different tissues.^2^ Epidemiologic studies revealed that the risk of cancer development inside the body increases with the disruption of the natural circadian rhythm, and thus with endogenous melatonin rhythms.^3^ In females, the disrupted natural circadian rhythm-induced breast cancer development is very common.^4^ Endogenous Mel modulates several signal transduction pathways associated with cell survival, migration, proliferation and apoptosis^5^ by counter-checking the redox status of cells, and thus acts as the checkpoints of the actions of several oncostatic factors.^6,7^ Moreover, Mel is a potent antioxidant with anti-inflammatory and antitumor properties. Because of this, it has been used as a therapeutic agent for treating diverse types of cancers.^8–11^ A cancer patient on Mel medication takes Mel into the body frequently due to its high oxidation affinity, short half-life (∼35–50 min) and slow dissolution properties. Mel gets metabolized easily, and is quickly eliminated from the patient's body.^12–16^ Mel conjugated with nanocarriers gets released slowly over a longer period, thereby reducing the dosage and improving its bioavailability to cancer cells.^17–21^ The different kinds of nanocarriers (such as chitosan–tripolyphosphate nanoparticles, polyethylene glycol microspheres (PEG), PLGA nanoparticles, gold nanoparticles (AuNPs), and others) have been used for the oral, intravenous and transdermal application of Mel.^19,22–24^ The limitations of previously reported nanocarriers include their multistep synthesis process, large size, higher cost of production, difficulty in scaling up, unexpected changes in pharmacokinetic behavior, toxic side products, accumulation in the body, heterogeneous phenomenon, poor drug loading and high burst release rate of the drug, among others.^25–30^

In recent years, fluorescent nanomaterials are high in demand for basic and industrial research due to their potential applications in the fields of bioimaging, sensing and drug delivery.^31–34^ Several materials and methods have been used to synthesize fluorescent nanomaterials.^35–37^ For last two decades, carbon quantum dots (CQDs) have been explored as a fluorescent material for different applications.^38,39^ However, toxic heavy metals (*e.g.*, cadmium, lead) are involved in the synthesis process of carbon quantum dots, which limits their widespread use in *in vivo* biological applications.^40,41^ Major concerns with present fluorescent materials are their size, low quantum yield, the generation of chemical waste, multi-step process and bio compatibility.^42^ These concerns have restricted the widescale biological application of available fluorescent materials especially *in vivo*, which has fueled the search for alternative nontoxic and environment-friendly nanoparticles for bioimaging and drug delivery.^43^

As an alternative, fluorescent nanodots synthesized from bio-molecules (such as DNA, nucleotides, and carbohydrates) have received increased interest due to their characteristics, like excellent biocompatibility, facile synthesis method, water solubility, high photostability, low cost, and higher life-time.^44–46^ In particular, based on their robust and tunable photoluminescence properties, nanodots are effectively employed in biomedical applications. Due to their beneficial aspect and an enormous range of applications, there is a necessity to devise efficient and simple routes for synthesizing other fluorescent nanodots of biological origin to improve their biological applications in *in vivo* conditions.

In the present study, biocompatible green-emitting protein nanodots (PNDs) having robust and tunable photoluminescence properties were synthesized from a common model protein lysozyme by a one-step hydrothermal method. The synthesis of PNDs as a drug carrier was optimized and used as a novel nanodelivery system for melatonin in breast cancer cells. They also enhanced the efficacy of the drug melatonin by a slow, sustained release in breast cancer cells. Furthermore, it has been exploited as bioimaging tools even at higher concentrations of melatonin, as no fluorescence quenching was observed. This makes it an ideal candidate for drug delivery and as a bioimaging tool in the biomedical field for *in vivo* study.

## 2. Materials and methods

### 2.1. Materials

Lysozyme (chicken egg white, lyophilized powder, single-chain 14 kDa with isoelectric point of 11.35), melatonin, glycine, hydrochloric acid, sodium dihydrogen phosphate, disodium hydrogen phosphate and sodium chloride were purchased from Sigma-Aldrich. All chemicals utilized were of analytical grade, and used without modifications.

### 2.2. Synthesis of PNDs and MPNDs

The synthesis of the PNDs was carried out *via* hydrothermal method.^47,48^ The homogenous solutions of lysozyme (40 mg ml^−1^) were prepared at two different pH values (pH-2 and pH-7 buffer were prepared using glycine-HCl buffer and Sorensen's phosphate buffer, respectively). The prepared solutions were transferred to a 200 ml Teflon cylinder with a steel autoclave, and heated for hydrothermal reaction at 200 °C for 15 hours. Furthermore, the solutions were cooled down and centrifuged at 10k rpm for 10 min. The supernatant was collected and filtered using the syringe filters (Axiva, 0.2 μm cutoff) to get the homogenous particle size. The filtered solution was collected and stored at room temperature for further studies. For *in vitro* study, MPND was prepared by adding a Mel working concentration (0.84 mM) to the PND working concentration (20 mg ml^−1^) at room temperature, and mixed vigorously by pipetting. The solution was incubated at room temperature for 1 h before *in vitro* administration.

### 2.3. Characterization of PNDs

The synthesized PNDs were thoroughly characterized. UV-Vis and photoluminescence measurements were carried out using an Eppendorf Biospectrometer and Fluorolog (Horiba), respectively. Fourier transform infrared (FT-IR) spectroscopy was performed over the wavenumber region of 500–4000 cm^−1^ with the resolution of 4 cm^−1^ using the Thermo Scientific Nicolet iD7 spectrometer. The transmission electron microscopy (TEM) image was obtained using a TECNAI-T-20 (FEI instrument) at 200 kV accelerating voltage. The zeta potential was measured by the Lite sizer 500 Anton Paar. Time-resolved fluorescence decay curves were obtained using a Horiba Jobin Yvon single-photon counting system with a 375 nm diode at a 1 MHz repetition rate and 1.3 ns pulse width. A Nikon LV100ND fluorescence microscope and Carl Zeiss 780 LSM laser scanning confocal microscopy system (Germany) were used for fluorescence microscopy. X-ray photoelectron spectroscopy was recorded using K-Alpha, Thermo Fisher Scientific equipped with a monochromatic Al Kα micro-focused X-ray source (100–4000 eV). The energy resolution was set at 0.1 eV at a pass energy of 1486.4 eV. The XPS spectra were analyzed using CASA XPS software. The background was first fitted with Shirley function and subtracted, and then the XPS peak was fitted with Gaussian Lorentzian lineshape functions.

The relative quantum yield of PNDs was evaluated with respect to quinine sulphate (Sigma Aldrich India, Ltd.). A standard curve was plotted between the integrated fluorescence intensity values with respect to the absorbance (<0.1) for different quinine sulphate (QS) solution concentrations prepared in 0.1 M H_2_SO_4_. A similar procedure was also followed for the PNDs suspension with deionized water acting as diluents. The slope, obtained from the linear fit standard curves, was used to calculate the quantum yield using eqn (1).1
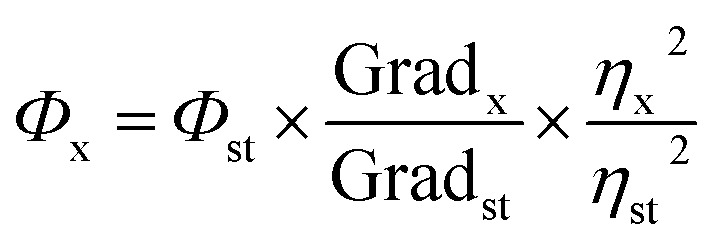
*Φ*_x_ = quantum yield of CQD, *Φ*_st_ = quantum yield of standard, Grad_x_ = gradient of curve plotted for CQD, Grad_st_ = gradient of curve plotted for standard, *η*_x_ = refractive index of the solvent used for preparing the CQD solution, *η*_st_ = refractive index of the solvent used for preparing the QS standard.

The quantum yield of QS has been estimated to be 0.54 in H_2_SO_4_ solution. The refractive index for 0.1 M H_2_SO_4_ was taken to be the same as that for DI water.

### 2.4. Cell culture

The human MG63 and MDA-MB-231 cancer cell lines were procured from the National Centre for Cell Science (NCCS) Pune, and cultured in Dulbecco's Modified Eagle Medium (DMEM) (High Glucose) with 10% FBS, 0.1 mM Minimum Essential Medium (MEM) Non-Essential Amino Acid (NEAA), 1% penicillin-streptomycin and 2 mM l-glutamine, as supplements. Cells were grown in T25 flasks with vented caps (Genetix) in a humidified atmosphere (Thermo Scientific, Heracellvios 160i) at 37 °C with 5% CO_2_. No contamination was observed in the cell line used for the experiments.

### 2.5. Cell viability assay

The cytotoxicity of the PNDs was determined by the 3-(4,5-dimethylthiazol-2-yl)-2,5-diphenyl tetrazolium bromide (MTT) assay in MG63 cells. MG63 cells were cultured in flat bottomed 96-well plates (Genetix) at 10^4^ cells per well for 24 h before the treatment. Cells were treated with complete media containing 10% FBS. To find the physiological dose at which the PNDs could be further used for bioimaging and drug delivery, the cells were plated into five groups (control and different dosages of PNDs, *i.e.*, 2 mg/100 μl, 4 mg/100 μl, 10 mg/100 μl and 20 mg/100 μl) for 24 h.

To determine the working dosage of Mel in the breast cancer studies, we performed a dose-dependent study with six different concentrations (0.1–1 mM) of Mel in MDA-MB-231 cells.^49^ After the incubation time, the MTT assay was performed according to standard protocol.^25,43^ The cytotoxicity was measured by determining the change of color formed due to formazan from 3-(4,5-dimethylthiazol-2-yl)-2,5-diphenyl tetrazolium bromide salt. The absorbance of the colored medium was measured at 595 nm using a microplate reader (instrument), and the percent viability was calculated by taking the viability of the control cells as 100%.

To evaluate the cytotoxic effect of Mel alone and MPNDs on the cell proliferation, the MTT assay was performed in MDA-MB-231 cells by treating with Mel and MPNDs, along with no treated control group after an incubation period of 24 h, 48 h and 72 h.

### 2.6. Cell migration assay

The cell migration capability was evaluated by wound closure assay. MDA-MB-231cells were seeded into 6 well plates for 24 h, and a vertical wound was made down through the cell monolayer using a 200 μl sterile micro tip by pressing firmly against the top of each well. The culture media containing cell debris from each well was removed gently without disturbing the cells, and treated with the derived concentration. In the control group, only complete DMEM media was added. Images were taken at an interval of 2 h, starting from the treatment time (0 h) up to 6 h. The quantitative analysis of the collective cell migration assay was performed by measuring the wound closure area using the following formula:Wound closure% = [(*A*_*t*_ = 0 h − *A*_*t*_ = Δ*h*)/*A*_*t*_ = 0 h] × 100%*A*_*t*_ = 0 h is the area of the wound measured immediately after scratching (*t* = 0 h), and *A*_*t*_ = Δ*h* is the area of the wound measured at *h* hours after the scratch is performed.^50^

### 2.7. Cellular uptake studies

One day prior to treatment, MG63 and MDA-MB-231 cells were seeded at 6 × 10^3^ cells per well on the cover slides, which are placed in the 24 well culture plate. The MG63 cells were then treated with 20 mg of pH-2 PNDs and pH-7 PNDs for 24 h at 37 °C. Subsequently, the MDA-MB-231 cells were treated with Mel and Mel-loaded pH-7 PNDs for 24 h at 37 °C. At 1 : 5000 concentration of DAPI, nuclear stain was added to the cells after 24 h of treatment. Finally, the images were captured by the fluorescence microscope and confocal microscopy system.

### 2.8. Statistical analysis

All statistical analyses were performed with a one-way analysis of variance (ANOVA) test and the results are represented as the mean ± SEM.

## 3. Results and discussion

### 3.1. Synthesis and characterization of the synthesized PNDs

**Fig. 1:**
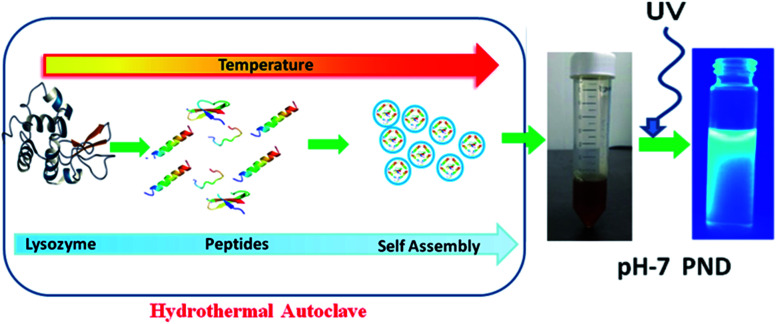
Schematic presentation of the synthesis of PNDs using the hydrothermal method.

To synthesize a novel drug delivery carrier with enhanced biological efficacy, protein nanodots were synthesized using a common model protein lysozyme. We demonstrate that these PNDs have excellent fluorescent property, and are biocompatible. In addition, the presence of different functional groups makes them an ideal candidate for biomaging and as a drug delivery carrier. The overall synthesis process and mechanism of formation of PNDs are schematically presented in Fig. 1. Above the melting temperature, the native structure of the protein deforms, leading to the formation of the small peptide strands. A further increase in temperature starts to break the peptide strands in the carbonaceous core nanostructure form with a functional activity *via* self-assembly.^51^ A previous report suggested that the temperature and the time duration in the hydrothermal technique play a significant role in the formation of the nanodots by controlling the particle size, which further enhances photoluminescence.^52,53^ For lower temperatures and short duration of heating, the organic molecule may not undergo appreciable carbonization of the material to produce fluorescence.^54^ So, we synthesized our PNDs at high temperature (200 °C) and longer duration (15 h). Above 70 °C, lysozyme completely melt at all pH ranges, but the conformation of the folded protein does depend on the pH value.^55^ To understand the effect of the conformation of the folded protein on the properties of the synthesized PNDs, the PNDs were synthesized at two different pH values (pH-2 and pH-7). During the hydrothermal process, native proteins at both pH values were heated at 200 °C for 15 h in order to disrupt the non-covalent bonds responsible for stabilizing the lysozyme. These PNDs were then extensively characterized using spectroscopic, optical and microscopic techniques.

**Fig. 2:**
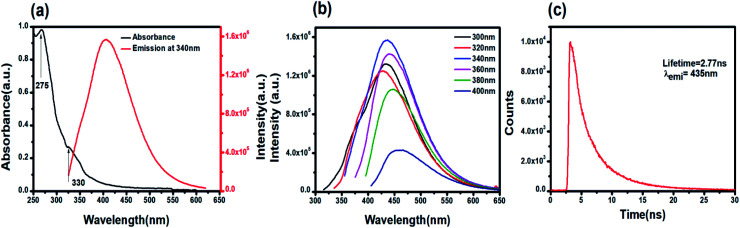
Optical characterization of PNDs: (a) UV-Vis absorption and emission spectra (*λ*_ex_ = 340 nm) of an aqueous dispersion of PNDs. (b) Excitation wavelength-dependent emission of PNDs. (c) Time-resolved PL decay curves of PND (*λ*_em_ = 435 nm).

The UV-Vis absorbance spectrum reveals the maximum absorption in the UV-blue region with two prominent absorbance peaks at 275 nm and 330 nm (Fig. 2a). The absorbance at 275 nm is ascribed to the π–π* electronic transition of C=C and the n–π* electronic transition of C=O present on the PNDs surface.^56,57^ The broad absorption extending between 330–350 nm is due to the presence of excited states of the functional groups on the surface.^58,59^ One of the most remarkable characteristics of PNDs is their intrinsic photoluminescence property, which has an excitation-dependent fluorescence spectrum with maximum fluorescence intensity found when excited at 340 nm (Fig. 2b). The wavelength-dependent emission in the nanodots can be attributed to the non-uniform size distribution of the PNDs and the presence of various surface functional groups, like carboxyl, hydroxyl and other oxygen species. Using eqn (1), the relative quantum yield of PNDs was calculated. It was found to be ∼14% using quinine sulfate as a reference in 0.1 M H_2_SO_4_.^60^ The fluorescence lifetime of PNDs at room temperature was measured with an average lifetime of 2.77 ns by fitting with a *R*^2^ value of 0.995 (Fig. 2c), where *τ*_1_ = 2.23 ns and *τ*_2_ = 5.94 ns were obtained. The multiexponential emission decay may arise from the rapid band gap transitions between different discrete states, or may also indicate the fast radiative recombination of multiple exciton species contributed by carboxyl, hydroxyl and other oxygen species.^44^ It has been reported that the higher doping of nitrogen in nanodots usually leads to a longer lifetime.^61^ A larger life time at the excited energy level makes PNDs an excellent material for cell imaging and fluorescent-dependent sensing.

**Fig. 3:**
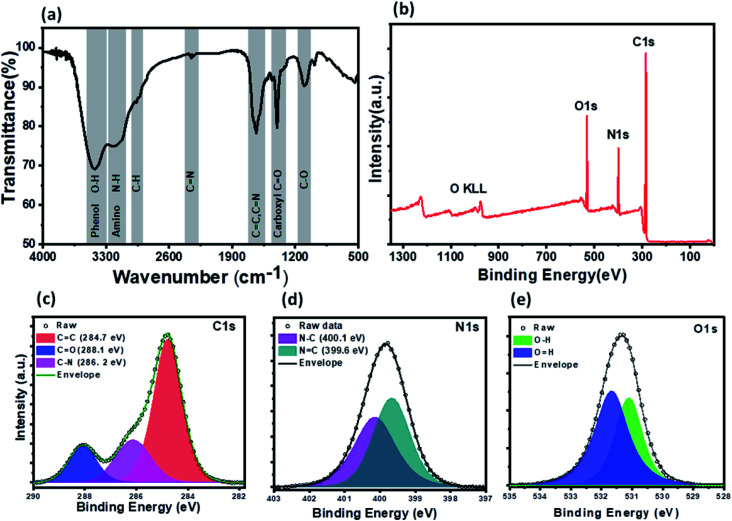
Spectroscopic characterization of PNDs: (a) FTIR spectrum of PND. (b) XPS survey spectrum. (c–e) High-resolution XPS spectra of C1s, N1s and O1s. The XPS peaks have been deconvoluted into several components.

XPS and FTIR studies were conducted to gain information about the chemical composition and functional groups on the surface of PNDs. The bands at 3418 cm^−1^ and 3186 cm^−1^ are due to the stretching vibrations of the O–H bond of the phenolic group and N–H bond of the amine (–NH_2_) group, respectively (Fig. 3a). The presence of a large number of O–H and N–H groups lends to the hydrophilicity to PNDs. The broad peak in the range of 1500–1700 cm^−1^ is composed of the stretching vibration of the C=C bonds, C=N bonds and N–H bond of the amine groups. The presence of the graphitic nitrogen bonded to three carbon atoms (C=N) is indicative of the nitrogen doping of the carbon core of PNDs. The peaks at around 1404 cm^−1^ and 1109 cm^−1^ are the stretching vibration of the C=O bond carboxylic groups and C–O bonds.^62^ The characteristic amide I (stretching of C=O) and amide II band (bending of the N–H and stretching of the C–N group) of the protein is not observed at all, indicating that all of the lysozyme has decomposed to form nanodots. The XPS survey scan of the PNDs shows that the PNDs are mainly composed of carbon, oxygen and nitrogen (Fig. 3b). The high-resolution C1s (Fig. 3c) spectra has been deconvoluted into three components, corresponding to the C=O group (288 eV), C–N group (286 eV) and C–C (284.8 eV). The N1s spectrum (Fig. 3d) has been fitted with two peaks at 399.6 and 400.3 eV, which are assigned to the N–C and N=C groups, respectively. The high resolution O1s (Fig. 3e) spectrum contains a peak at 531 eV & 531.6 eV due to the presence of the O–H and C=O groups, respectively. The higher percentage of nitrogen than oxygen in the PND surface leads to the high fluorescence intensity at very low concentration in aqueous solution. Both XPS and FTIR measurements show that there are oxygen-containing functional groups and nitrogen-containing amino group present. PNDs synthesized at pH-2 showed a similar pattern (Fig. S2–S5†), which suggests that both PNDs have similar physical properties and functional groups. However, the differences in the ratio of the functional groups present on the surface and defects are possible reasons for the different maximum excitation wavelengths.

**Fig. 4:**
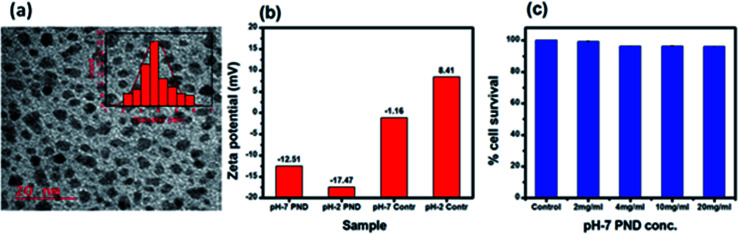
(a) TEM of PNDs, histogram of the particle size distribution is shown in the inset. (b) Zeta potential of the PNDs. (c) Concentration-dependent cell viability of PND for 24 h.

Transmission electron microscopy (TEM) was employed to estimate the size and morphology of the PNDs. The PND diameter lay within the range of 3–5 nm with an average diameter of around 3.5 nm (Fig. 4a). The zeta potential measurement of the synthesized PNDs at pH-2 and pH-7 showed that PNDs possess a negative surface zeta potential of −17.4 mV and −12.5 mV, respectively (Fig. 4b), showing the stability of PNDs in solution owing to the electrostatic repulsion between the individual PNDs.^63^

### 3.2. *In vitro* cell cytotoxicity study of PNDs

The physical characterization of the PNDs suggested that it is a good fluorescence carrier for bioimaging. On the other hand, the presence of a wide range of functional groups on its surface makes it an efficient nanocarrier with higher drug loading efficiency. To be an efficient drug carrier for biological applications, the PNDs should be biocompatible with no/minimal cellular toxicity. We first studied whether the PNDs of both pH values (pH-2 and pH-7) are non-toxic to the cells using MTT assay. At pH = 2, the MTT assay for different concentrations of PNDs showed cell death (3–21%) in MG63 at all studied concentrations (Fig. S4a†). One of the possible reasons could be that the cell has a low survival rate at acidic pH. On the contrary, when we analyzed the cytotoxicity of pH-2 PNDs in a pH-7 buffer solution, the cells showed 99% survival (Fig. S4a†). This shows the importance of the pH in the cell cytotoxicity study. At physiological pH (pH-7.4), the cells are more viable. In a similar way, we analyzed the toxicity of pH-7 PNDs using different concentrations (Fig. 4c), and the result showed only 3–0.6% cell death, which was very low as compared to the pH-2 PNDs. Thus, we can say that the PNDs synthesized at pH-7 are less cytotoxic.

### 3.3. PNDs as a fluorescent tool for bioimaging studies

**Fig. 5:**
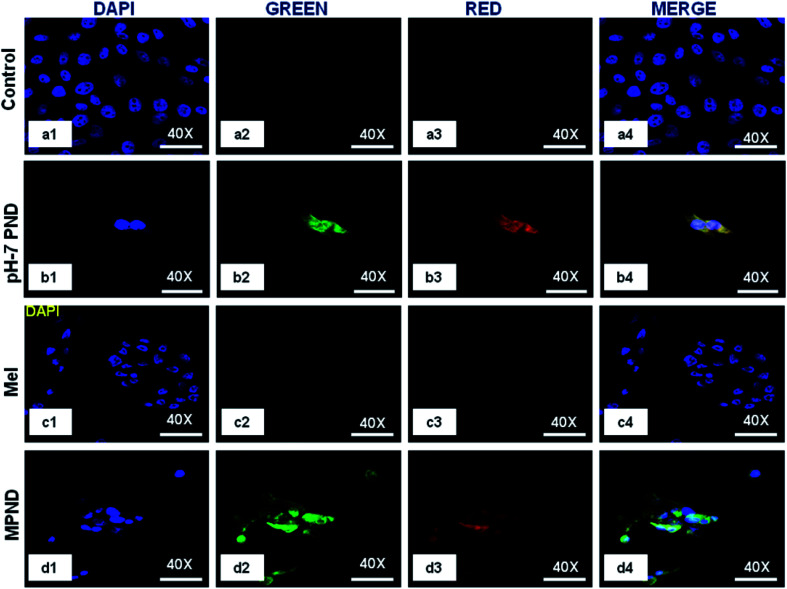
Confocal images of the breast cancer cell control (a1–a4), and treated with pH-7 PNDs (20 mg ml^−1^: (b1–b4)), only Mel (0.84 mM: (c1–c4)) and Mel-pH-7 PNDs (MPNDs) (0.04 mM + 20 mg ml^−1^ pH-7 PNDs: (d1–d4)).

In recent years, the nucleus has been considered as a diagnostic biomarker for the pre-monitoring of diseases, like cancer, and in cellular stress conditions due to changes in its morphology, like nuclear fragmentation, nuclear blebbing and nuclear size.^16^ PNDs (pH-2 and pH-7) confirmed its permeability through confocal imaging in both cytoplasm and nucleus, and thus enhanced the possibility of nuclear drug delivery applications (Fig. 5(b1–b4)). We also studied the intensity level of the fluorescence of both PNDs using different concentrations. The results showed high fluorescence at 20 mg ml^−1^ concentration in both pH-2 and pH-7 PNDs, whereas at 4 mg ml^−1^ (low) concentration, the pH-2 PNDs showed no florescence from the cells. In contrast, the pH-7 PNDs still showed minimum florescence (Fig. S5(a1–a4), (b1–b4), (c1–c4) and S6(a1–a4), (b1–b4), (c1–c4)).† Therefore, for further studies, the pH-7 PNDs were used at 20 mg ml^−1^ concentration as a fluorescent nanocarrier for the bioimaging and drug delivery of Mel (Fig. 5(d1–d4)).

### 3.4. Physical characterization of Mel-loaded PNDs (MPNDs)

**Fig. 6:**
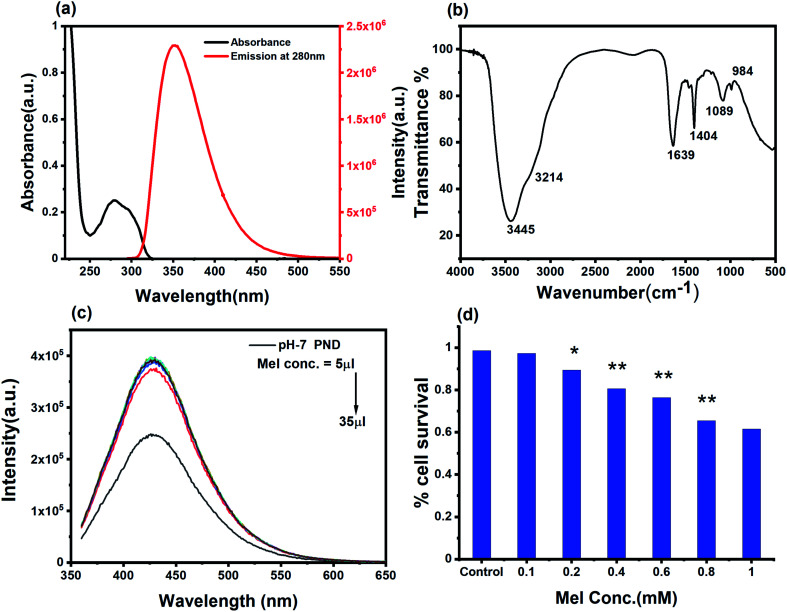
(a) UV-Vis and emission spectra of melatonin (emission spectra was measured with excitation wavelength), (b) FTIR spectrum of pH-7 PNDs and Mel-pH-7 PNDs (MPNDs), and (c) emission spectra of Mel-pH-7 PNDs (MPNDs) at increasing concentration of Mel, (d) cell viability study of dose-dependent Mel for 24 h.

The optical characterization of Mel shows an absorbance peak at 275 nm. The excitation and emission spectra showed that both excitation wavelengths (260 nm and 313 nm) correspond to the same emission range with a peak position at 356 nm (Fig. 6a). The pH-7 PND and Mel conjugate (MPNDs) are stable in the water suspension for several months without a change in their optical properties. By analyzing the absorbance peak of Mel and the emission peak of PNDs, we can conclude that no fluorescence resonance energy transfer (FRET) phenomenon is happening between PND and Mel.^64^ The FTIR spectrum of a pure MEL standard showed major peaks of functional groups at 3306 and 3260 cm^−1^ (N–H bending and C–N stretching), 1492 and 1550 cm^−1^ (aromatic –C=), 1630 cm^−1^ (C=O), 1180 and 1217 cm^−1^ (–C–O), and the complex formed with PNDs showed a small shift in the peak positions (Fig. 6b).^25^ The FTIR study shows that the functional groups present on the PNDs surface, mainly double bonds (C=O, C=C and phenolic groups), can interact with the aromatic ring of Mel with dipole-induced dipole forces and form a complex, which can facilitate charge transfer from the donor to acceptor. The complex shows a higher increase in the fluorescence intensity at very low concentration of Mel, and as we increase the concentration of Mel in the system, the intense fluorescence remains constant (Fig. 6c). The Mel interaction with the PNDs provides new states and defects for the charge transfer, and enhance the photoluminescence intensity.^64,65^ The UV and PL analysis of the complex shows that PNDs can be a very good carrier for the bioimaging and drug delivery of Mel for further *in vitro* studies.

### 3.5. Studying the therapeutic bio-efficacy of Mel-loaded PNDs (MPNDs)

**Fig. 7:**
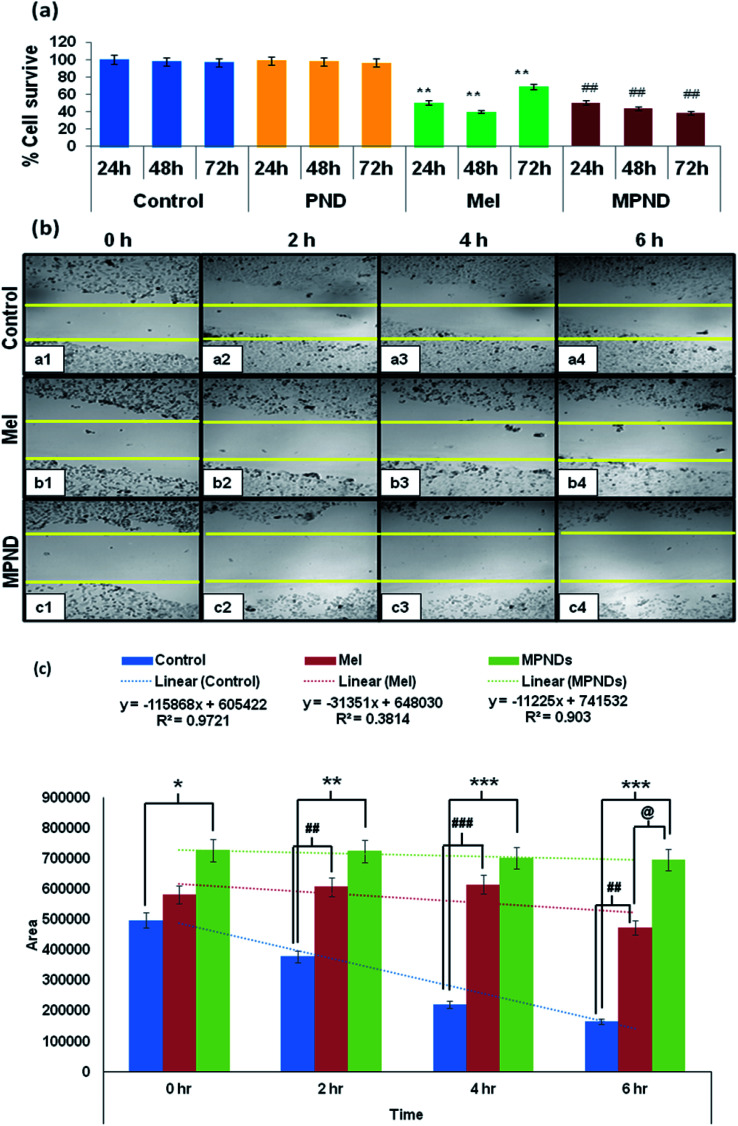
(a) Cell proliferation analysis with Mel and MPNDs by MTT assay. ** showed the significance level of *p* < 0.001 between the control and Mel, whereas ## showed the significance level of *p* < 0.001 between Mel and MPND. (b) Cell migration study with Mel (b1–b4) and MPNDs (c1–c4). (c) Histogram showing the wound closure% within the group comparison. * denotes the significant changes between the control and MPNDs groups; # denotes the significant changes between the control and Mel groups, and @ denotes the significant changes between Mel and MPNDs groups.

In order to study and understand the biological efficiency of MPNDs for breast cancer treatment, we first measured the IC_50_ of the Mel drug alone in MDA-MB231 cells, which was 0.84 mM, and this concentration was further used for all drug treatments in the present study. Mel was then loaded in PNDs, and MPNDs were prepared at 0.84 mM concentration (Fig. 6d). The IC_50_ values of Mel has been shown in the parabolic and linear graphs in Fig. S4(b and c),† respectively. MDA-MB231 cells were treated with Mel and MPNDs separately, and incubated for 24 h. The physical characterization of Mel showed negligible self-fluorescence at the cellular level; thus, the cells treated by Mel only showed no fluorescence (Fig. 5(c1–c4)). On the other hand, PNDs and MPNDs allowed for the visualization of morphological changes after treatment in confocal imaging (Fig. 5(d1–d4)). The cell proliferation analysis *via* MTT assays after 24 h, 48 h and 72 h incubations with Mel only and MPNDs separately showed a significant decrease in the cellular mortality in both groups for up to 48 h of treatment when compared to the control and ‘only PND’ treated cells. Interestingly, after 72 h of treatment, the Mel-treated cells showed less cell mortality (31.33%), whereas the MPNDs still showed high cell mortality (61.53%). From these results, we may conclude that the MPNDs might have helped in the slow release of the drug, making it more effective for a longer period of time, *i.e.*, 72 h (Fig. 7a). Various nano-carriers have already been reported for the effective drug delivery of Mel in cancer therapies. The majority of them possess self-toxicity towards the cancer cells, which enhances the mortality efficacy of Mel in *in vitro* cancer treatment with time.^25,66^ However, this self-toxicity of the nano carriers is also capable of harming other parts of the body, aside from cancerous cells. Importantly, our synthesized PND was non-toxic. Thus, the retained cell mortality of the MDA-MB231 cells for at least 72 h was totally due to the slow release of the drug Mel from MPND.

Cell migration is involved in several pathological processes, such as tumor invasion, neoangiogenesis and metastasis. To study the efficacy of MPND as compared to Mel alone on cell migration, we performed the *in vitro* wound healing assay. We found that the breast cancer cell migration becomes slower with Mel treatment, and also cell death was observed from the wound site at the time interval of 4 h and 6 h in comparison to the control cells. In comparison with only Mel treatment, the MPNDs-treated cells showed no cell migration at the time interval of 2 h and 4 h, and very small cellular progression towards the wound at 6 h was observed. Simultaneously, the higher cell death was also observed in the MPNDs-treated cells at the wound sites at 2 h, 4 h and 6 h (Fig. 7b and c). These results support the previous views of melatonin in breast cancer treatment with the added benefit that MPNDs lowers the limitation of the half-life of Mel, which enhances the efficiency of Mel in lowering the cell proliferation and cell migration.^67,68^

## 4. Conclusion

In the present study, we have reported an efficient, simple and cost-effective method for the synthesis of protein nanodots (PNDs) at two different pH values using lysozyme as a protein source. The synthesized PNDs were thoroughly characterized using TEM, FTIR, UV-Vis, and XPS for morphological and chemical analysis. Further, the synthesized PNDs were used as a carrier for the loading of melatonin, and the synthesized MPNDs nano drug delivery system was characterized. They showed remarkable aqueous stability and photoluminescence property. The application of developed MPNDs was studied on breast cancer cells. MPNDS showed higher cellular uptake, more cytotoxicity and invasion properties compared to free melatonin. Thus, a novel nano drug delivery system for the melatonin breast cancer cell was successfully synthesized and applied as an efficient bioimaging and drug delivery system.

## Conflicts of interest

There are no conflicts to declare.

## Supplementary Material

RA-011-D0RA08959A-s001
