# Supplementary material for: Synthesis and characterization of novel protein nanodots as drug delivery carriers with an enhanced biological efficacy of melatonin in breast cancer cells
Source: RSC Adv. 2021 Mar 1;11(16):9076–85. doi: 10.1039/d0ra08959a (PMC8695413; doi:10.1039/d0ra08959a)
Supplement: RA-011-D0RA08959A-s001 [file RA-011-D0RA08959A-s001.pdf]

Electronic Supplementary Material (ESI) for RSC Advances.
This journal is © The Royal Society of Chemistry 2021

Supplementary Information

Synthesis and Characterization of Novel Protein Nanodots as Drug Delivery Carrier with Enhanced Biological Efficacy of Melatonin in Breast Cancer Cells

Kanchan Yadav[1], Megha Das[2], Nurul Hassan[3], Archana Mishra[4], Jayeeta Lahiri[3], Ashutosh Kumar Dubey[5], Sanjeev Kumar Yadav[2], Avanish Singh Parmar[1]*

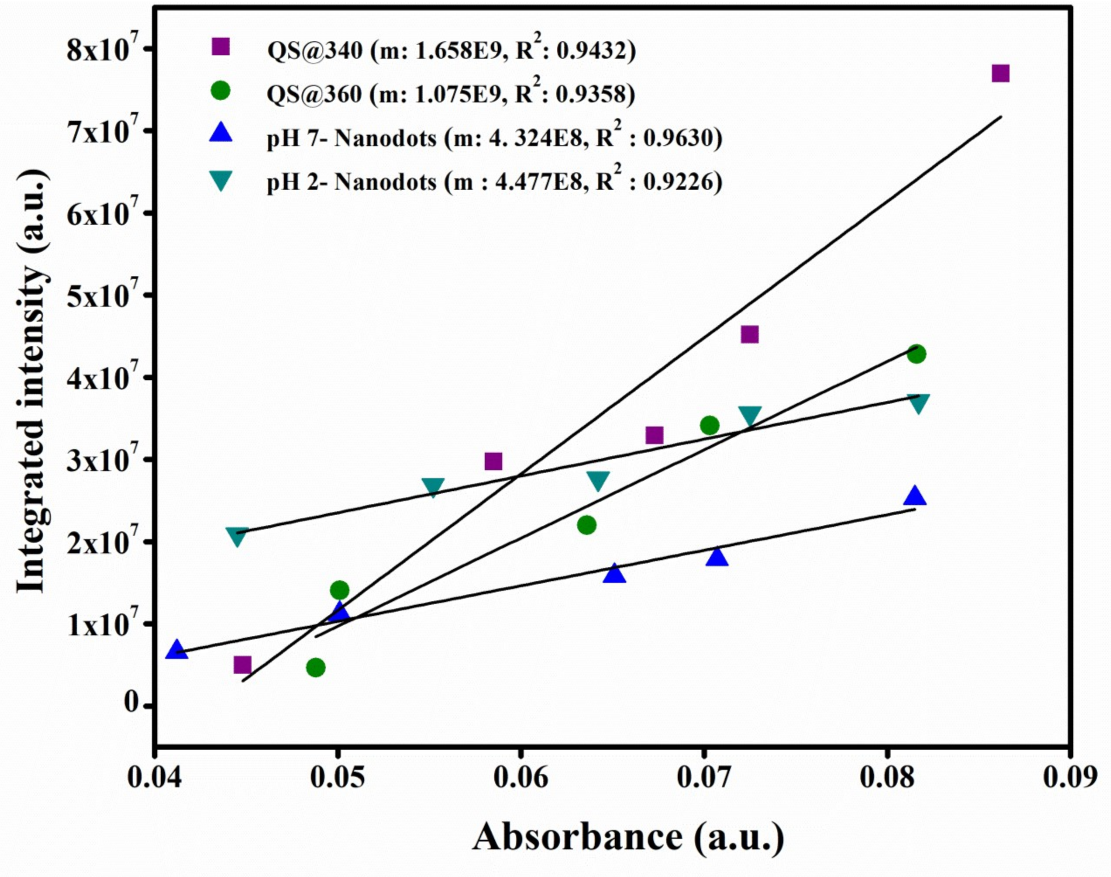

Fig. S1: Linear plot representing integrated emission intensity Vs. Absorbance

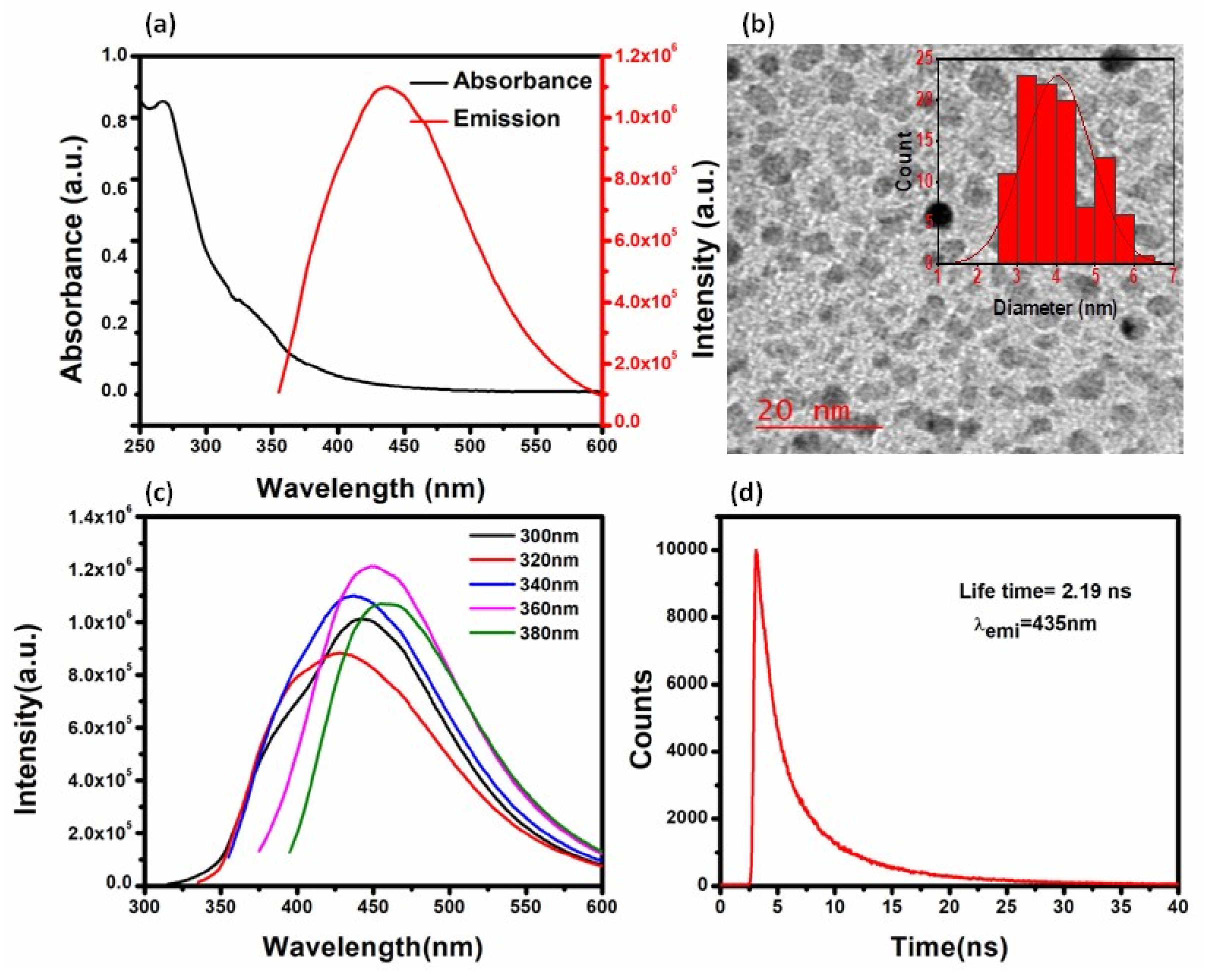

Fig. S2: Optical characterization of pH-2 PND (a) UV-Vis spectra and Emission spectrum at 340nm (b) TEM images of pH-2 PND; Histogram shows the particle distribution (c) Wavelength dependent emission spectra (d) Time resolved PL decay curves of pH-2 PND (λ_{em} =435 nm)

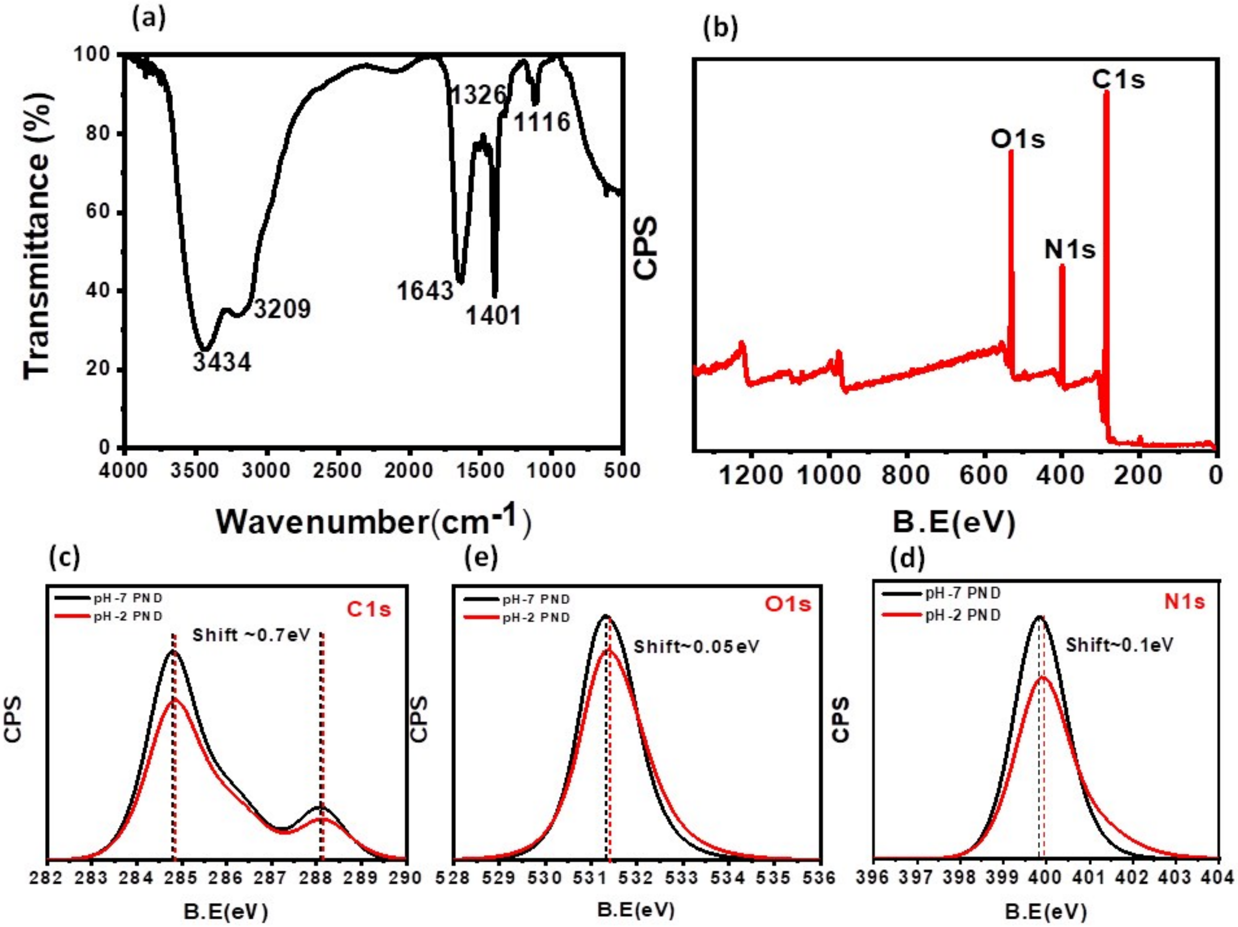

Fig. S3: (a) FTIR spectrum of pH-2 PND (b) Full scan XPS spectrum of pH-2 PND (c)- (d) shows the comparative shift in peak position of C1s, O1s and N1s of pH-7 and pH-2 PND.

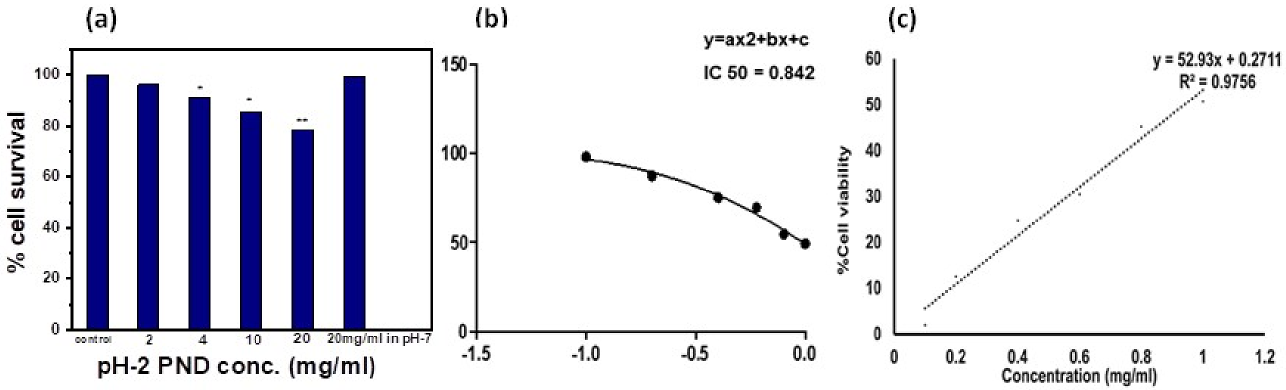

Fig. S4: (a) Concentration dependent cell viability of pH-2 PND for 24hr. * and ** showed the significance level of $p<0.05$ and <0.001 between control and after PND of different concentration administrated groups respectively. (b)- (c) Parabolic graph and linear graph of Mel dose dependent cell survival for IC50 value analysis.

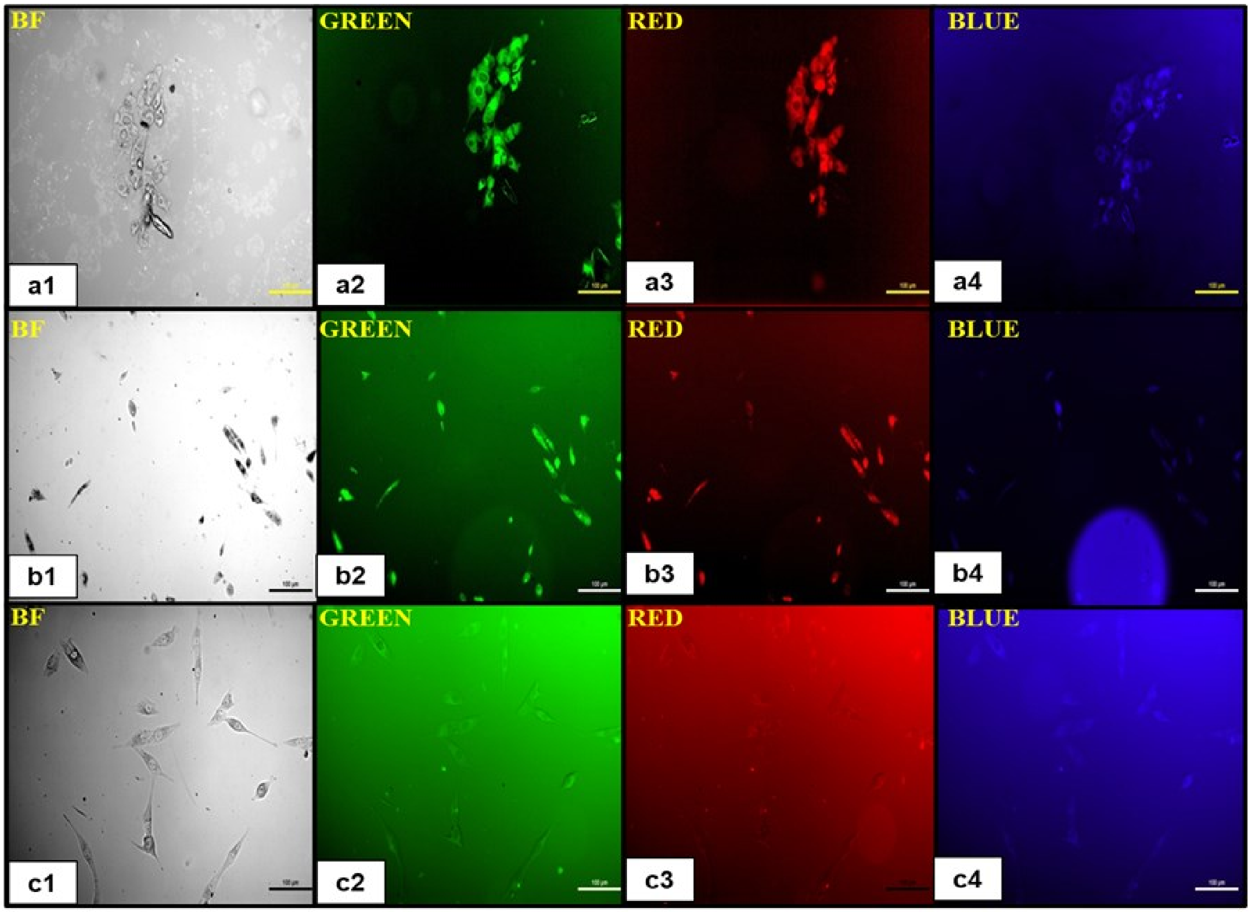

Fig S5 : Fluorescence imaging of pH-2 PNDs in different concentrations (a1-a4: 20mg/ml; b1-b4: 10mg/ml; c1-c4: 4mg/ml) in MDA-MB-231 breast cancer cells

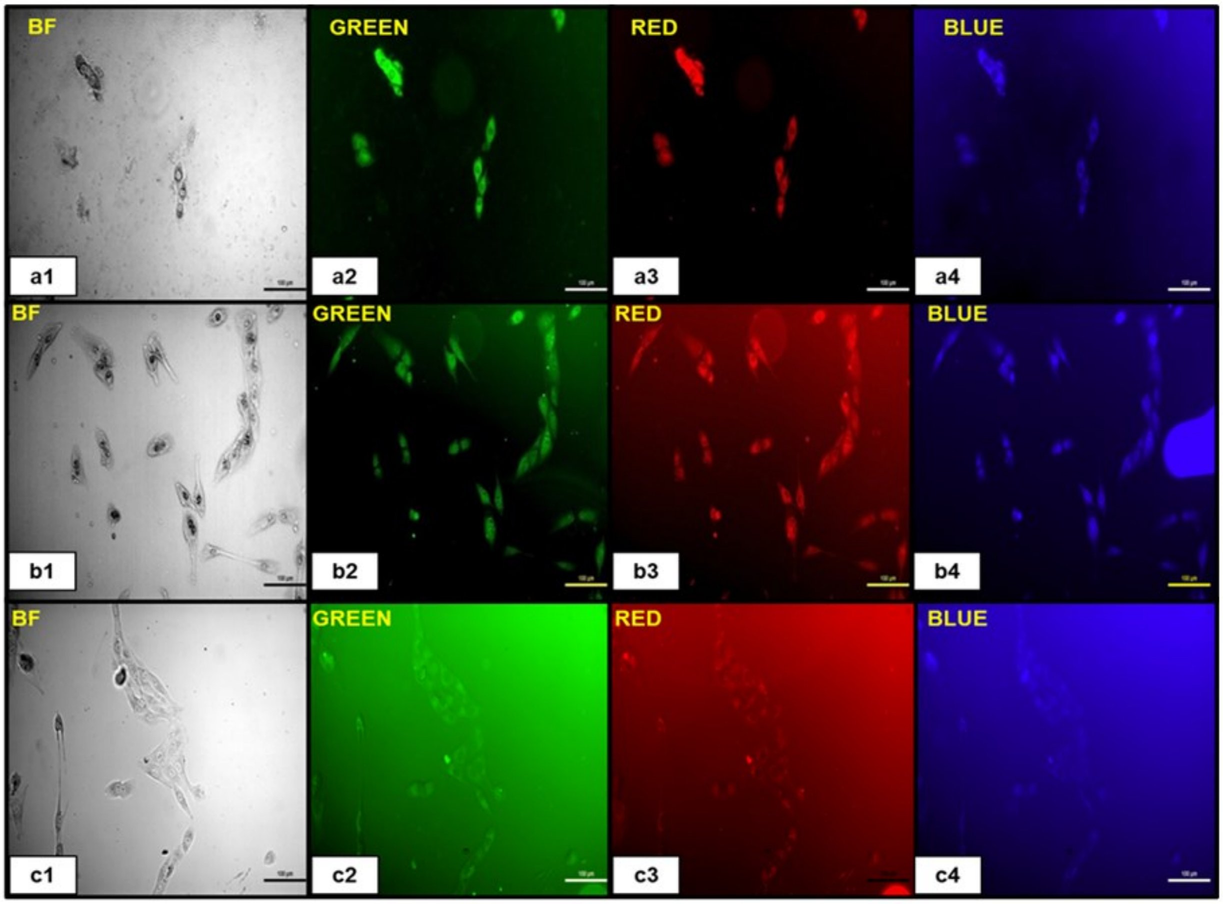

Fig. S6: Fluorescence imaging of pH-7 PNDs in different concentrations (a1-a4: 20mg/ml; b1-b4: 10mg/ml; c1-c4: 4mg/ml) in MDA-MB-231 breast cancer cells
